# Parameters Influencing the Growth of ZnO Nanowires as Efficient Low Temperature Flexible Perovskite-Based Solar Cells

**DOI:** 10.3390/ma9010060

**Published:** 2016-01-19

**Authors:** Alex Dymshits, Lior Iagher, Lioz Etgar

**Affiliations:** Institute of Chemistry Casali Center for Applied Chemistry, The Hebrew University of Jerusalem, Jerusalem 91904, Israel; alexanderdym@gmail.com (A.D.); lioriagher@gmail.com (L.I.)

**Keywords:** ZnO nanowires, flexible solar cells, growth rate, precursor concentration, perovskite

## Abstract

Hybrid organic-inorganic perovskite has proved to be a superior material for photovoltaic solar cells. In this work we investigate the parameters influencing the growth of ZnO nanowires (NWs) for use as an efficient low temperature photoanode in perovskite-based solar cells. The structure of the solar cell is FTO (SnO_2_:F)-glass (or PET-ITO (In_2_O_3_·(SnO_2_) (ITO)) on, polyethylene terephthalate (PET)/ZnAc seed layer/ZnO NWs/CH_3_NH_3_PbI_3_/Spiro-OMeTAD/Au. The influence of the growth rate and the diameter of the ZnO NWs on the photovoltaic performance were carefully studied. The ZnO NWs perovskite-based solar cell demonstrates impressive power conversion efficiency of 9.06% on a rigid substrate with current density over 21 mA/cm^2^. In addition, we successfully fabricated flexible perovskite solar cells while maintaining all fabrication processes at low temperature, achieving power conversion efficiency of 6.4% with excellent stability for over 75 bending cycles.

## 1. Introduction

The photovoltaic field has recently experienced a breakthrough with hybrid organic-inorganic perovskite used as a light harvester in solar cells. The hybrid perovskite is simple to deposit, has a large absorption coefficient, and a long diffusion length—the reason for the high power conversion efficiency (PCE) of 20.1% [1] in such a short period of time. The perovskite solar cell can be used in mesoporous or planar architecture [2]. In addition, it has been reported that the perovskite does not necessarily require hole transport material [3].

The photoanode of mesoporous perovskite solar cells is usually an n-type semiconductor (metal oxide) nanostructure that allows electron injection from the perovskite to the metal oxide. Moreover, in the case of perovskite solar cells, a scaffold metal oxide such as Al_2_O_3_ can be used where electron injection is not possible [4]. The most common metal oxide used in perovskite solar cells is mesoporous TiO_2_ [5,6]. However, TiO_2_ has low electron mobility, which could create unbalanced charge transport in the perovskite. A possible alternative uses nanostructure ZnO, which is an n-type semiconductor with an energy gap (E_g_) of 3.37 eV at 300 K and a higher electron mobility than TiO_2_ [7,8].

A few reports present the possibility of working with one-dimensional ZnO in perovskite solar cells [9,10,11,12]. However, the photovoltaic (PV) performance of the ZnO-based cells is still lower than the TiO_2_-based cells. Hagfeldt *et al.* have reported that the reason for the low PCE is due to more recombination in the case of one-dimensional ZnO [10]. Furthermore, it is necessary to optimize and study the growth conditions of one-dimensional ZnO in order to increase the PCE of these ZnO nanowires perovskite solar cells.

We performed a detailed study on the growth of ZnO nanowires (NWs), investigating the parameters that influence the growth rate and the NW diameter. Once the growth conditions were better understood, we were able to fabricate ZnO NWs CH_3_NH_3_PbI_3_ perovskite solar cells that achieved high PCE (over 9%) with current density over 21 mA/cm^2^. In addition, we successfully fabricated flexible perovskite solar cells while maintaining all fabrication processes at low temperature, achieving power conversion efficiency of 6.4% with high stability over seventy-five bending cycles.

## 2. Results and Discussion

**Figure 1:**
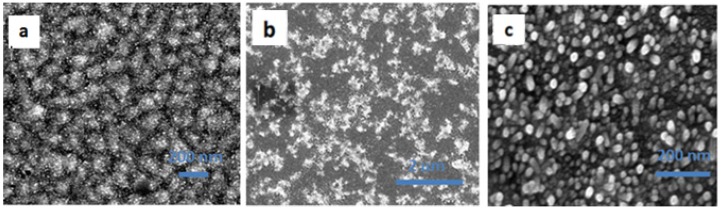
Top view SEM images of ZnAc seed layer at (**a**) 0 min; (**b**) 5 min; (**c**) 10 min. Scale bar is 200 nm for images A and C, and 2 μm for image B.

In this work, using hydrothermal growth, we grew ZnO NWs, which function as the photoanode in perovskite-based solar cells. The growth parameters (growth time and precursors concentration) were studied to achieve the optimal conditions for use of ZnO NWs (as the photoanode) in the perovskite solar cells. The ZnO NWs require a seed layer for the initiation of growth; in this study, zinc acetate dehydrate (ZnAc) is used as the seed layer. Previous reports [13] showed that the annealing temperature required for the ZnAc seed layer is 350 °C to achieve a crystalline seed layer. We decided to maintain the processes at low temperature, in order to fabricate flexible devices, as discussed below; therefore, the ZnAc seed layer was annealed to only 120 °C, resulting in an amorphous seed layer as shown in the X-ray diffraction (XRD) spectra in Figure S1. Figure 1 shows scanning electron microscopy (SEM) top view images of the ZnAc seed layer at 0 min, 5 min, and 10 min growth of the ZnO NWs. It can be observed that the ZnO NWs growth began randomly on the ZnAc seed layer surface. Already after 10 min, most of the electrode is covered by short ZnO NWs with a length of 48–67 nm, depending on the growth rate, which is influenced by the concentration of the precursor (as discussed below). In addition, it can be observed that the crystallinity of the ZnAc is not mandatory for the growth of ZnO NWs.

**Figure 2:**
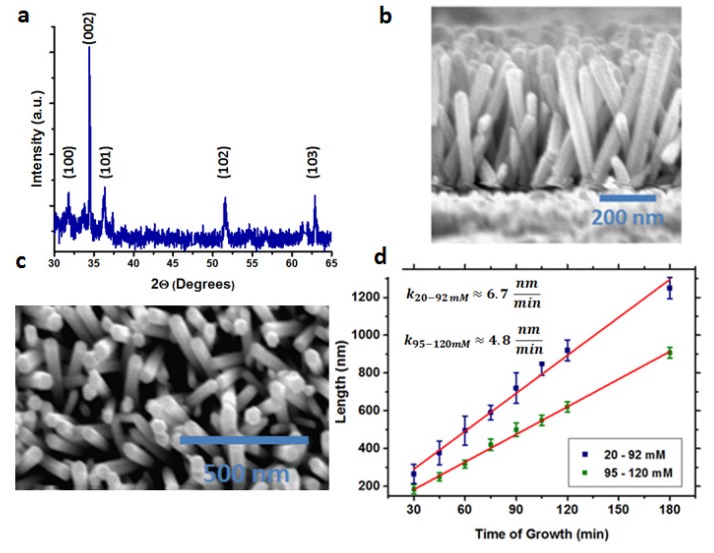
(**a**) XRD of the ZnO nanowires (NWs); (**b**) SEM cross section of the ZnO NWs grown at 90 mM and 75 min; (**c**) SEM top view of the ZnO NWs grown in the same conditions as in Figure 2b; (**d**) The NWs length as a function of the growth time for the two different concentration regimes.

The solution for the ZnO NWs growth contains zinc nitrate hexahydrate and hexamethylenetetramine (HMTA) dissolved in deionized water. Growth took place at 90 °C for different concentrations of precursors and different growth times. Figure 2a presents the XRD spectra of the ZnO NWs, demonstrating highly oriented ZnO NWs. The dominant (002) peak shows that the c-axis is perpendicular to the FTO substrate [14,15]. The length of the ZnO NWs is controlled by the growth time, while the diameter is not affected when the growth time is changed. The diameter changes with the precursor concentration. Two concentration regimes can be observed from Figure 2d, resulting in two different growth rates—regime 1: 20–92 mM and regime 2: 95–120 mM, corresponding to growth rates of k = 6.7 nm/min and k = 4.8 nm/min, respectively. The growth rate in regime 2 (high concentrations) is slower than in regime 1 (low concentrations), due to the fact that at high concentrations there is an excess of precursors, which causes the reaction to occur in the solution before arriving at the substrate. As a result, the growth rate is slower. Figure 2b,c shows SEM cross-section and top view images, respectively, of the ZnO NWs grown at 75 min and 90 mM.

**Figure 3:**
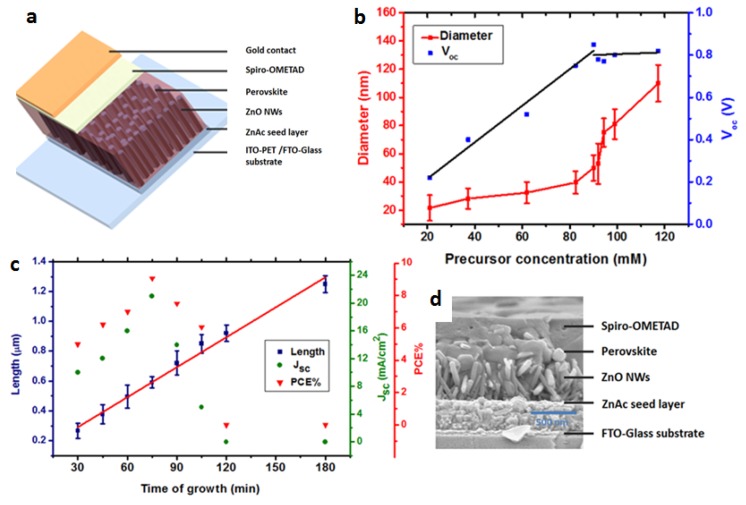
(**a**) The CH_3_NH_3_PbI_3_ perovskite ZnO NWs solar cell structure; (**b**) The ZnO NWs length and the PV parameters as a function of the growth time at precursor concentration of 90 mM; (**c**) The change in the ZnO NWs diameter and the open circuit voltage (V_oc_) as a function of the precursor concentration at 75 min growth time; (**d**) SEM cross section of the CH_3_NH_3_PbI_3_ perovskite ZnO NWs perovskite solar cell.

The diameter of the ZnO NWs is altered by changing precursor concentration, as indicated in Figure 3b. If the precursor concentration is increased, the diameter increases. It can be observed that from 20 to 92 mM there is a slow increase in the NWs diameter (linear increase); this was also reported by Park *et al.* [16] for these concentrations. However, from 95 to 120 mM, the diameter increased by approximately 60 nm. This increase corresponds to the two different concentration regimes also observed in Figure 2d. It can be concluded that in terms of growth conditions, ZnO NWs can be grown in two different precursor concentration regimes, resulting in different diameters and different growth rates. Importantly, when using the high concentration regime (95–120 mM), the NWs growth is dense (see Figure S2 in the Supplementary Materials), preventing the perovskite from penetrating through the entire ZnO NWs film.

Using this knowledge, perovskite solar cells based on ZnO NWs as the photoanode were fabricated. Figure 3c shows the solar cell structure composed of FTO glass/ZnAc seed layer/ZnO NWs/CH_3_NH_3_PbI_3_ perovskite/spiro-OMeTAD/Au. The ITO-PET (polyethylene terephthalate) substrate mentioned in Figure 3c is used for the flexible low temperature perovskite-based solar cells (discussed below). An SEM cross-section image of the perovskite ZnO NWs solar cell (on FTO glass substrate) is shown in Figure 3d.

To understand the influence of the ZnO NWs length and diameter on the photovoltaic performance, we used a precursor concentration of 90 mM, which, according to Figure 2d and Figure 3b, results in a grow rate of 6.7 nm/min and a diameter of *ca.* 50–55 nm. As indicated, a higher precursor concentration results in dense growth of the ZnO NWs, which prevents penetration of the perovskite. Moreover, it can be observed that the open circuit voltage (V_oc_) of the cell increases with the precursor concentration, but at 90 mM and above, the V_oc_ does not change as a function of the precursor concentration (Figure 3b). According to previous reports, the maximum V_oc_ achieved for ZnO NWs photoanode in perovskite solar cells is around 0.8 V [7,9]; it is suggested that the ZnO surface suffers from a high rate of recombination, which reduces the V_oc_. The change in the photovoltaic (PV) parameters as a function of the growth time is observed in Figure 3a. From 30 to 75 min, the PV parameters increase, while for a longer growing time, the PV parameters decrease. Increasing the growing time results in longer NWs, although when the NWs are too long (over ≈700 nm), the perovskite cannot completely cover the NWs, resulting in direct contact of the ZnO NWs with the hole transport material (spiro-OMeTAD) and the gold electrode, reducing PV performance. Therefore, growth time of 75 min gives the highest PV performance.

**Figure 4:**
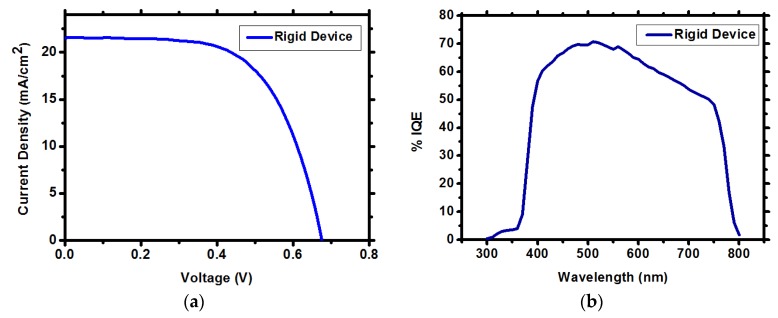
(**a**) IV curves of the ZnO NWs CH_3_NH_3_PbI_3_ perovskite solar cell on rigid substrate; (**b**) IQE spectra of the corresponding rigid solar cell.

Fabricating the CH_3_NH_3_PbI_3_ perovskite ZnO NWs solar cell (on FTO glass) using the optimal conditions discussed (90 mM, 75 min growth time at 90 °C) results in the highest PV performance. Table 1 and Figure 4a show the PV parameters and the IV curves of the best CH_3_NH_3_PbI_3_ perovskite ZnO NWs solar cell on rigid substrate with current density of 21.5 mA/cm^2^, Fill Factor (FF) of 62%, V_oc_ of 0.67 V and efficiency of 9.06%. The ZnO-based solar cells show small hysteresis, as presented in Figure S3. The internal quantum efficiency (IQE) is presented in Figure 4b for the rigid substrate having typical shape for CH_3_NH_3_PbI_3_ perovskite-based solar cells covering the whole visible region 400–780 nm wavelength. The average power conversion efficiency of the rigid solar cells, based on 13 cells, is 7.5% ± 0.6%. The histogram of the solar cells’ efficiency is presented in Figure S4 of the supporting information.

**Table 1:** Photovoltaic Parameters of ZnO/CH_3_NH_3_PbI_3_/Spiro-OMeTAD Solar Cells.

Solar Cell Substrate	Jsc (mA/cm^2^)	FF (%)	V_OC_ (V)	Efficiency (%)	Average (%)
Rigid Device (FTO-Glass)	21.56	62.3	0.675	9.06	7.5% ± 0.6
Flexible Device (ITO-PET)	14.42	64.7	0.684	6.39	5.6% ± 0.6

**Figure 5:**
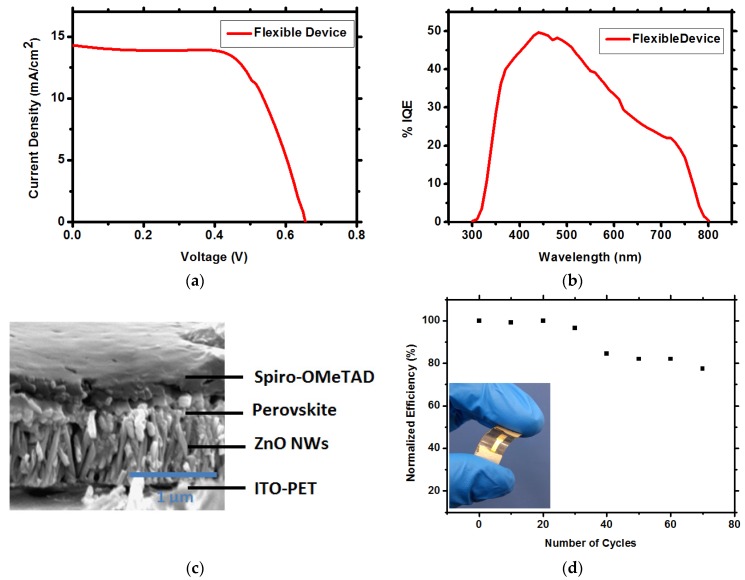
(**a**) IV curves of the ZnO NWs CH_3_NH_3_PbI_3_ perovskite solar cell on flexible substrate; (**b**) IQE spectra of the corresponding flexible solar cell; (**c**) SEM cross section of the flexible ZnO NWs CH_3_NH_3_PbI_3_ perovskite solar cell; (**d**) Stability of the flexible solar cell under bending. The bending radius is 1.2 cm. Inset: image showing typical, flexible ZnO NWs CH_3_NH_3_PbI_3_ perovskite solar cell.

Fabrication processes of the solar cells were performed at low temperature (ZnAc deposition, ZnO NWs growth, and CH_3_NH_3_PbI_3_ perovskite deposition). No annealing steps at temperatures over 120 °C were required, enabling the use of flexible substrate, PET coated with ITO. The conditions for the NWs growth on flexible substrate are similar to the best conditions used for the rigid substrate, 90 mM, 90 °C, and 90 min growth time. The flexible ZnO NWs CH_3_NH_3_PbI_3_ perovskite-based solar cell demonstrates V_oc_ of 0.684V, FF of 64%, J_sc_ of 14.4 mA/cm^2^, with PCE of 6.39% (Table 1 and Figure 5a). The average PCE of the flexible device, based on 10 cells (each sample contains a single pixel, *i.e.*, single solar cell), is 5.6% ± 0.65%. The IQE of the flexible solar cell (Figure 5b) is lower than the rigid solar cell, due to difference in the current density. The SEM cross section of the flexible solar cell is presented in Figure 5c, clearly showing the layers and the penetration of the CH_3_NH_3_PbI_3_ perovskite through the ZnO NWs film. Figure 5d shows the stability of the flexible solar cell under bending (radius of bending was 1.2 cm). High stability can be observed for over 75 cycles, during which the efficiency dropped less than 20% from its initial value.

## 3. Experimental

### 3.1. ZnO NWs Growth

The substrate of the rigid device is SnO_2_: F (FTO) conducting glass (15 Ω∙cm, Pilkington, UK). The substrate of the flexible device is In_2_O_3_·(SnO_2_) (ITO) on PET, polyethylene terephthalate film, purchased from Sigma (Rehovot, Israel). The substrates were cleaned with deionized water and dried under a flow of clean air. The growth method of ZnO NWs followed previous publications [11,14]. The seed layer was prepared by dissolving 35 mM of zinc acetate dehydrate (ZnAc)(Zn(CH_3_COO)_2_·2H_2_O, 98%, Sigma, Rehovot, Israel) in ethanol and stirred at 80 °C for 2 h. The solution of ZnAc was spin casted on the substrates at a spinning rate of 2000 rpm for 30 s, followed by heating at 90 °C to remove the solvent. This process was repeated two times, and finally the ZnAc seed layer was annealed at 120 °C. The solution for growing ZnO NWs was prepared by dissolving equal molar weight of zinc nitrate hexahydrate (Zn(NO_3_)_2_·6H_2_O, Sigma, 98%, Rehovot, Israel) and hexamethylenetetramine (HMTA, Sigma, 99%, Rehovot, Israel) in twice-deionized water (TDW). The precursor concentration of the growth solution varied from 20 to 120 mM; initially, the solution was preheated for 1 h at 90 °C and the temperature kept constant throughout the procedure. The ZnAc deposited on the FTO/ITO substrates was immersed in the growth solution for different periods of time. Finally, the ZnO NWs film was rinsed with deionized water several times and dried at 100 °C for 20 min.

### 3.2. Solar Cell Fabrication

CH_3_NH_3_I was synthesized as described in the literature [17,18]. The CH_3_NH_3_PbI_3_ perovskite was deposited on top of the ZnO NWs by a two-step method described elsewhere [19]. First, PbI_2_ was dissolved in Dimethylformamide (DMF) at a concentration of 1 M and dropped onto the layer of ZnO NWs and spin coated at 2000 rpm for 15 s. Next the cell was annealed at 80 °C for 30 min. In the second step, the cell was dipped into methylammonium solution (0.063 M in isopropanol) for 30 s, following the annealing at 80 °C for 30 min.

The spiro-OMeTAD solution was prepared according to the previous reports [20,21]. 36 mg of 2,2′,7,7′-tetrakis-(N,N-di-4-methoxyphenylamino)-9,9′-spirobifluorene (spiro-OMeTAD) was dissolved in 0.5 mL of chlorobenzene. Then 13.2 µL of bis (trifluoromethane) sulfonimide lithium salt in acetonitrile (520 mg/mL), 14.5 µL of tris(2-(1 H-pyrazol-1-yl)-4-*tert*-butyl pyridine)-cobalt(III) tris(bis (trifluoromethylsulfonyl)imide)) in acetonitrile (300 mg/mL) and 9.6 µL of 4-*tert*-butilpyridine were added to the spiro-OMeTAD solution as additives. The spiro-OMeTAD solution was deposited above the layer of perovskite by spin coating 40 µL at 4000 rpm for 30 s.

#### 3.2.1. Scanning Electron Microscopy (SEM)

The images were obtained using Sirion HR-SEM of FEI (Field Emission Instruments, Eindhoven, The Netherlands). The measurement conditions were 5–10 kV at various magnifications, as seen on the data bar of the images.

#### 3.2.2. Solar Simulator

Photovoltaic measurements were made on a New Port system, composed of an Oriel I–V test station using an Oriel Sol3A simulator (Irvine, CA, USA). The solar simulator is class AAA for spectral performance, uniformity of irradiance, and temporal stability. The solar simulator is equipped with a 450 W xenon lamp. The output power is adjusted to match AM1.5 global sunlight (100 mW/cm^2^). The spectral match classifications are IEC60904-9 2007, JIC C 8912, and ASTM E927-05. I–V curves were obtained by applying an external bias to the cell and measuring the generated photocurrent with a Keithley model 2400 digital source meter (Keithley, Culver, CA, USA). The sweep direction of the measurement was forward to reverse with maximum reversed voltage of −0.1 V and maximum forward voltage of 1.2 V. The cells were measured without light pre-treatment. The voltage step and delay time of photocurrent were 10 mV and 40 ms, respectively. Photovoltaic performance was measured by using a metal mask with an aperture area of 0.05 cm^2^.

#### 3.2.3. Incident Photon to Current Efficiency (IPCE)

Oriel IQE-200 (Irvine, CA, USA) was used to determine the monochromatic incident photon-to-electric current conversion efficiency. Under full computer control, light from a 150 W xenon arc lamp was focused through a monochromator in the 300–1800 nm wavelength range onto the photovoltaic cell under the test. The monochromator was incremented through the visible spectrum to generate the external quantum efficiecny (EQE(λ)) as defined by EQE (λ) = 12 400 (J_sc_/λφ), where λ is the wavelength, J_sc_ is the short-circuit photocurrent density (mA/cm^2^), and φ is the incident radiative flux (mW/cm^2^). IQE = EQE/(1-Reflectaion-transmission) which was measured at the same system as the EQE measurements.

#### 3.2.4. X-ray Diffraction

Measurements were performed using the D8 Advance diffractometer (Bruker AXS, Karlsruhe, Germany) with a secondary graphite monochromator, 2° soller slits and a 0.2 mm receiving slit. XRD patterns within the range 5° to 60° 2θ were recorded at room temperature using CuKα radiation (λ = 1.5418 Å) with the following measurement conditions: a tube voltage of 40 kV, a tube current of 40 mA, step-scan mode with a step size of 0.02° 2θ, and a counting time of 1–3 s per step. At the GIXRD (grazing incidence X-ray diffraction), the value of the grazing incidence angle was 2.5°.

## 4. Conclusions

In this work, we presented ZnO NWs perovskite-based solar cells on rigid and on flexible substrates. The growth time and precursor concentration of the ZnO NWs were investigated. We observed that the NWs diameter is affected by the precursor concentration and not by the growth time. In addition, two different growth regime rates were recognized—the high concentration regime shows a lower growth rate than the low concentration regime. Optimizing the ZnO NWs growth conditions enables achieving PCE of 9.06% with current density of 21.5 mA/cm^2^ for the ZnO NWs perovskite solar cells. Moreover, flexible ZnO NWs perovskite solar cells were fabricated, demonstrating excellent stability over 75 bending cycles with PCE of 6.4%. This investigation shows that ZnO NWs can be used as an efficient alternative to low temperature photoanode in perovskite-based solar cells.

## Supplementary Materials

The following are available online at www.mdpi.com/1996-1944/9/1/60/s1.
