# Supplementary material for: Parameters Influencing the Growth of ZnO Nanowires as Efficient Low Temperature Flexible Perovskite-Based Solar Cells
Source: Materials (Basel). 2016 Jan 19;9(1):60. doi: 10.3390/ma9010060 (PMC5456534; doi:10.3390/ma9010060)
Supplement: Supplementary file 1 [file materials-09-00060-s001.pdf]

Supplementary Materials: Parameters Influencing the Growth of ZnO Nanowires as Efficient Low Temperature Flexible Perovskite-Based Solar Cells

Alex Dymshits, Lior Iagher and Lioz Etgar

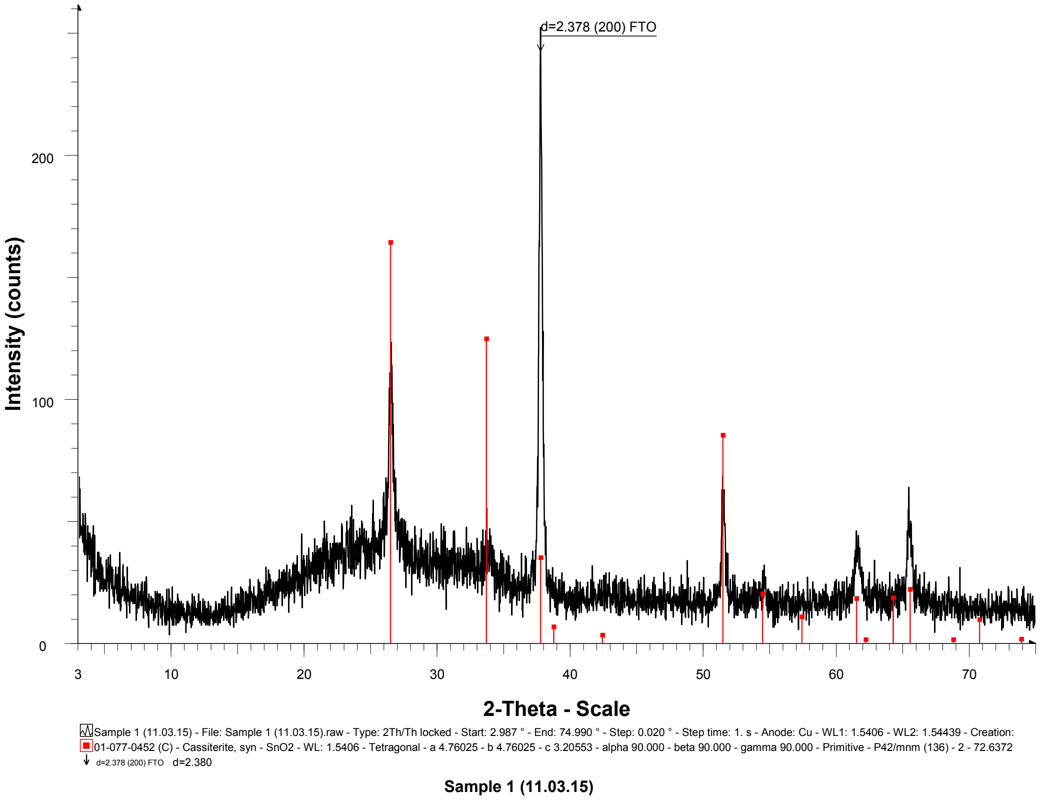

Figure S1. XRD spectra of the ZnAc seed layer. The only peaks observed in this spectrum are related to the FTO coating. Experimental parameter: 01-077-0452, Cassiterite, syn, SnO_2; Crystallite Size (Scherrer): 287.9, A; System: Tetragonal; Space group: P42/mnm (136); Cell parameter: (Initial → Final): a: 4.75520 → 4.76025; c: 3.19920→3.20553.

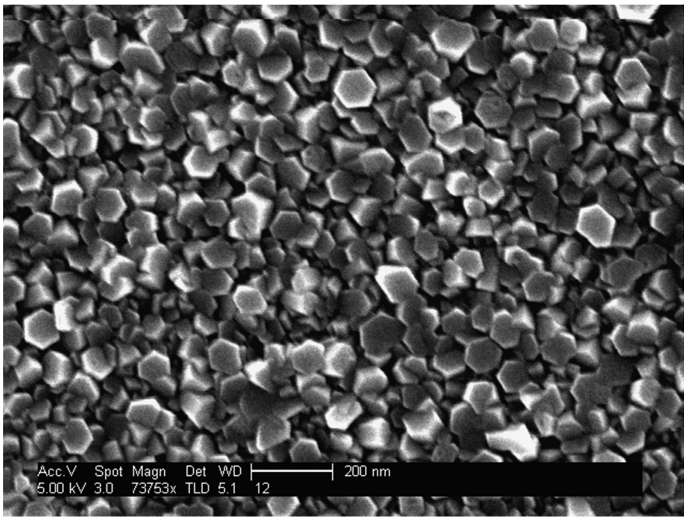

Figure S2. SEM top view of ZnO NWs grown at concentration of 110 mM.

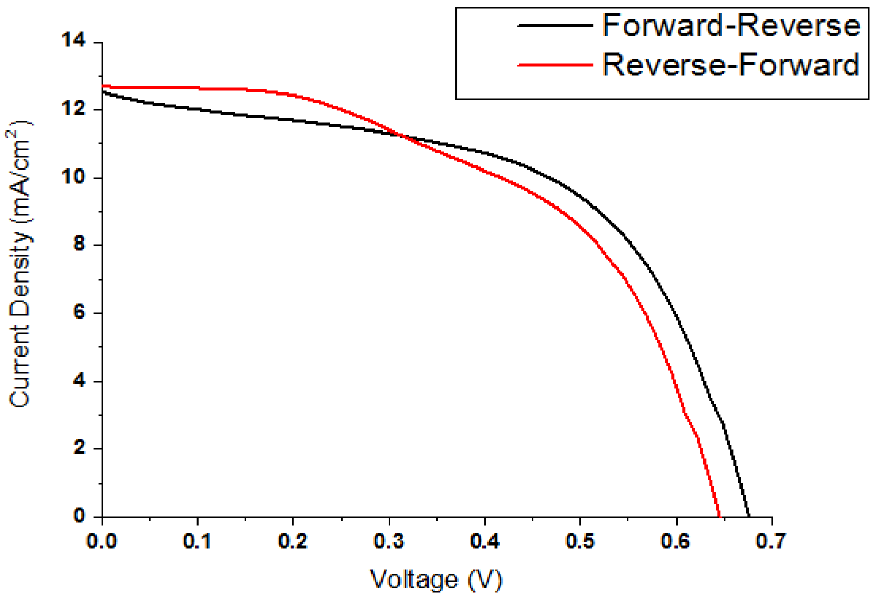

Figure S3. Example of forward to reverse and reverse to forward scan of the ZnO rigid solar cell. The Hysteresis index is −0.007 with scan rate of 0.032 V/s.

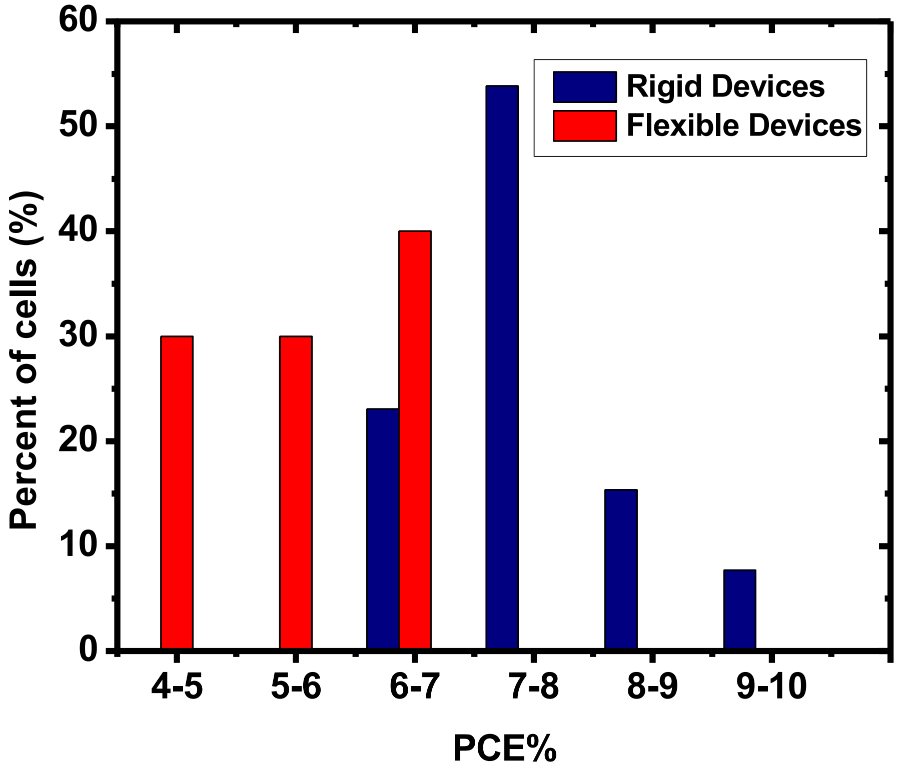

Figure S4. Histogram of the solar cells efficiency prepared in this study.
